# Electrical Characterization of a Double-Layered Conductive Pattern with Different Crack Configurations for Durable E-Textiles

**DOI:** 10.3390/mi11110977

**Published:** 2020-10-30

**Authors:** Tomoya Koshi, Ken-ichi Nomura, Manabu Yoshida

**Affiliations:** Sensing System Research Center (SSRC), National Institute of Advanced Industrial Science and Technology (AIST), 1-1-1 Higashi, Tsukuba, Ibaraki 305-8565, Japan; k-nomura@aist.go.jp (K.-i.N.); yoshida-manabu@aist.go.jp (M.Y.)

**Keywords:** conductive pattern, e-textiles, crack configuration, meandering metal pattern, conductive ink pattern, laser cutting, screen printing, tensile test

## Abstract

For the conductive patterns of electronic textiles (e-textiles), it is still challenging to maintain low electrical resistance, even under large or cyclic tensile deformation. This study investigated a double-layered pattern with different crack configurations as a possible solution. Patterns with single crack growth exhibit a low initial resistance and resistance change rate. In contrast, patterns with multiple crack growth maintain their conductivity under deformation, where electrical failure occurs in those with single crack growth. We considered that a double-layered structure could combine the electrical characteristics of patterns with single and multiple crack growths. In this study, each layer was theoretically designed to control the crack configuration. Then, meandering copper patterns, silver ink patterns, and their double layers were fabricated on textiles as patterns with single and multiple crack growths and double-layered patterns, respectively. Their resistance changes under the single (large) and cyclic tensile deformations were characterized. The results confirmed that the double-layered patterns maintained the lowest resistance at the high elongation rate and cycle. The resistance change rates of the meandering copper and silver ink patterns were constant, and changed monotonically against the elongation rate/cycle, respectively. In contrast, the change rate of the double-layered patterns varied considerably when electrical failure occurred in the copper layer. The change rate after the failure was much higher than that before the failure, and on the same order as that of the silver ink patterns.

## 1. Introduction

Electronic textiles (e-textiles) have been developed by many research groups [1,2,3,4,5,6,7,8,9]. One of the e-textile applications is seamless healthcare monitoring under various situations [10,11,12,13]; therefore, the durability of e-textiles against mechanical deformation is crucial. For durable e-textiles, conductive patterns fabricated on textiles that maintain low electrical resistance even under large or cyclic tensile deformation are key components. In previous studies, to achieve such patterns, researchers have investigated new materials such as conductive yarns [14,15,16], conductive inks [17,18,19,20], and new structures, such as meander-patterned metal foils [13]. However, in general, conductive yarns and inks exhibit a higher initial resistance and resistance change rate against deformation than the meander-patterned metal foils. Moreover, electrical failure occurs easily in meander-patterned metal foils under deformation [21]. Thus, patterns maintaining low resistance under deformation are still challenging.

Therefore, this study investigated a double-layered structure of patterns exhibiting different crack configurations under tensile deformation, as a possible solution. The crack due to deformation results in a change in the resistance of the pattern. The crack size and density differ considerably depending on the materials, structures, and the fabrication process of the patterns. Patterns with different crack configurations show different electrical characteristics. A previous study [21] classified the crack configurations into two types: single and multiple crack growths. Single crack growth is often observed in patterns using metal foils with thicknesses of several micrometers or more on stretchable substrates, such as polydimethylsiloxane and polyurethane [22,23,24]. In many cases, the metal foil is patterned to a meandering shape, and a single large crack occurs at the apex of the meander. Multiple crack growth is often observed in patterns using very thin metal films (thickness of several tens of nanometers) [25,26,27] or stretchable conductive inks [28] on stretchable substrates. Many microcracks occur in the whole of the pattern, regardless of the shape of the pattern. Comparing these patterns, the patterns with the single crack growth exhibit lower initial resistance because of their lower material resistivity and thicker pattern thickness. In addition, they exhibit a lower resistance change rate against deformation. In contrast, patterns with multiple crack growth maintain their conductivity even under deformations where electrical failure occurs in patterns with singe crack growth. The reason is that the growth rate of multiple cracks against deformation is much slower. We considered that a double-layered structure could combine these electrical characteristics, and that the combined characteristics should maintain a low resistance even under deformation. That is, we considered that the layer with single crack growth should contribute to the low initial resistance and resistance change rate. Moreover, the layer with multiple crack growth should contribute to maintaining the conductivity even under deformation where electrical failure occurs in the layer with single crack growth.

In this study, each layer of the patterns was theoretically designed to control the crack configuration. Then, the patterns with single and multiple crack growths and their double layers were fabricated on textiles, and their resistance changes under the single (large) and cyclic tensile deformations were characterized.

## 2. Materials and Methods

### 2.1. Theoretical Design of Conductive Patterns

**Figure 1:**
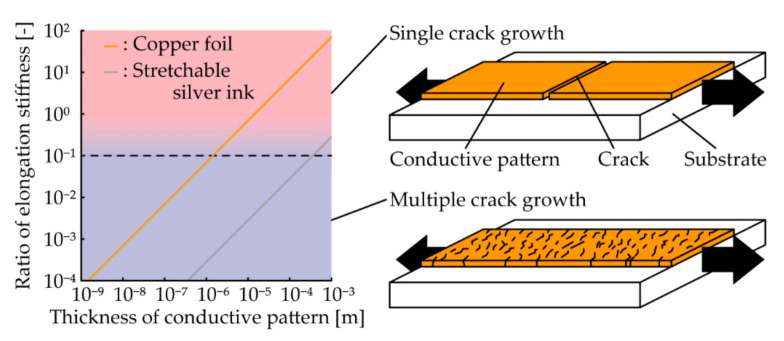
Calculation of the ratio of elongation stiffnesses of the pattern and substrate plotted against the pattern thickness.

A previous study [21] reported that the crack configuration is determined by the ratio of elongation stiffnesses of the conductive pattern and substrate. When the ratio is greater than approximately 0.1, single crack growth is observed in the pattern. In contrast, when the ratio is smaller than approximately 0.1, multiple crack growth is observed in the pattern. In this study, the ratio of the elongation stiffness was calculated to determine the materials and thicknesses of the patterns and substrates for each crack configuration. Figure 1 shows the ratio of elongation stiffness plotted against the pattern thickness using different conductive materials. The calculation was conducted using *σ*_u_*t*_c_/*E*_s_*t*_s_, where *σ*_u_, *t*_c_, *E*_s_, and *t*_s_ are the ultimate (breaking) stress of the pattern, thickness of the pattern, Young’s modulus of the substrate, and thickness of the substrate, respectively. A copper foil and stretchable silver ink were used in the calculation because they are typically used for conductive patterns. The ultimate stresses of the copper foil and silver ink are 0.2 GPa [29] and 0.8 MPa, respectively. For the substrate, we assumed plain weave cotton with a thickness of 0.22 mm and Young’s modulus of 13 MPa. In Figure 1, the ratios vary to 0.1 when the pattern thickness is 1.4 μm and 0.36 mm for the copper foil and silver ink, respectively. For the single crack growth, it is difficult to fabricate silver ink patterns with a thickness of more than 0.36 mm, because this thickness is very thick for the printing processes. Therefore, considering fabrication simplicity, we used an 18-μm-thick copper foil with a lamination process, which is often used for conventional printed circuit boards. In addition, we used a meandering shape for the copper foil, because copper foil itself does not have much stretchability. For multiple crack growth, the fabrication of a copper foil pattern with a thickness of less than 1.4 μm requires a complex process, such as evaporation or electroless plating with photolithography. Moreover, these processes cause thermal or chemical damage to the textile substrate. Therefore, considering fabrication simplicity, we used a several-tens-micrometers-thick silver ink formed by screen printing.

### 2.2. Fabrication of Conductive Patterns

**Figure 2:**
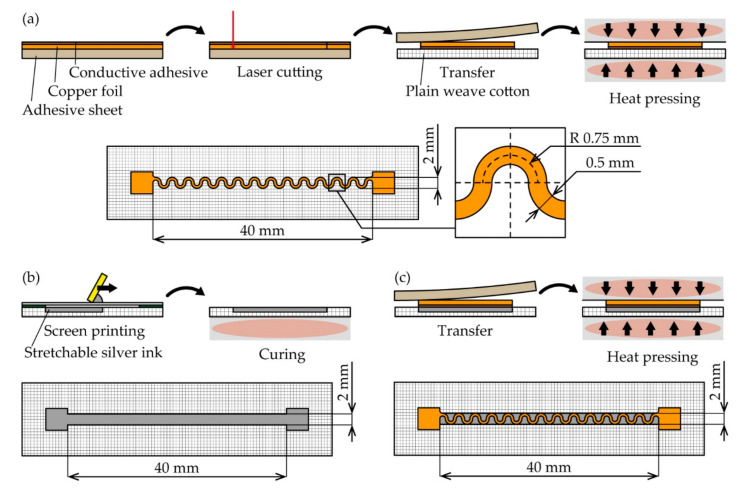
Fabrication processes and dimensions for (**a**) meandering copper (single crack growth); (**b**) silver ink (multiple crack growth); and (**c**) double-layered patterns (a combination of single and multiple crack growths).

The fabrication processes and dimensions of the conductive patterns with different crack configurations are shown in Figure 2a–c. In this study, for a pattern with single and multiple crack growth, meander-shaped copper and linear-shaped silver ink patterns were used, respectively. Their double-layered pattern was fabricated by transferring the meandering copper pattern onto the silver ink pattern with a soft conductive adhesive. For the textile substrate, commercially available plain weave cotton, with a thickness of 0.22 mm and Young’s modulus of 13 MPa, was used. The diameter and pitch of the yarns of the plain weave cotton were 0.2 and 0.3 mm, respectively. Each pattern was designed to fit a width of 2 mm and length of 40 mm, and the contact pads for external electrical connections were designed at the both ends of the patterns.

For the meandering copper patterns (Figure 2a), an 18-μm-thick copper foil (CU-18C, 3M Company, Saint Paul, MN, USA) was laminated on an adhesive sheet (CR09300-A3, Graphtec Co., Kanagawa, Japan). An acrylic-based soft conductive adhesive (0.04 mm in thickness) was coated on one side of the copper foil, and the foil was laminated with the conductive adhesive side up. Then, the conductive adhesive and copper foil were patterned to the meandering pattern using a laser cutting machine (8032 speedy360 flex, Trotec Laser, Marchtrenk, Austria). The unnecessary area of the copper foil was delaminated using a tweezer, and the patterned copper foil was transferred to the plain weave cotton. The adhesive force between the copper foil and the plain weave cotton caused by the conductive adhesive was stronger than that between the copper foil and the adhesive sheet, and therefore the foil could be transferred to the cotton. Finally, the patterned copper foil was heat-pressed at 120 °C and 3 MPa for 30 s using a heat-pressing machine (H300-05, As One Co., Osaka, Japan) to enhance the mechanical adhesion between the foil and the plain weave cotton. The width and radius of the meander shape were 0.5 and 0.75 mm, respectively. For the silver ink patterns (Figure 2b), a stretchable silver ink (SSP2801, Toyobo Co., LTD., Osaka, Japan) was printed on plain weave cotton by screen printing. The viscosity of the ink was 20.5 Pa·s at the shear rate of 100 s^−1^, and the ink was overprinted to control the thickness of the ink. The measurement of the ink thickness is described in Section 3.1. The printed ink was then heated at 120 °C for 30 min by a hot plate to cure the ink. For the double-layered patterns (Figure 2c), a copper foil patterned to the meandering shape was prepared, as shown in Figure 2a. A stretchable silver ink was also printed, as shown in Figure 2b. Subsequently, the copper pattern was transferred onto the printed ink pattern by the adhesion caused by the conductive adhesive on the copper pattern. The transferred copper pattern was heat-pressed at 120 °C and 3 MPa for 30 s to enhance the electrical and mechanical adhesion between the copper foil and the silver ink.

### 2.3. Measurement Setup

**Figure 3:**
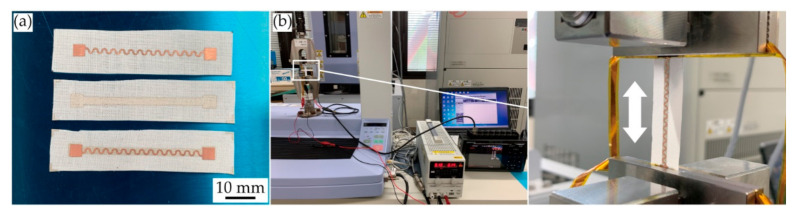
Optical images of (**a**) fabricated samples; and (**b**) measurement setup for single and cyclic tensile deformations.

Figure 3a,b show the measurement setup. Before the single and cyclic tensile deformations, the fabricated samples were observed with an optical microscope and a scanning electron microscope (SEM) (VHX-D510, Keyence Co., Osaka, Japan). Then, the sample was mounted on a tensile testing machine (AGS-X, Shimadzu Co., Kyoto, Japan), and single (large) or cyclic tensile deformation was applied to the sample. For the single tensile deformation, the deformation speed was 8.3 mm/min, and the elongation rate was gradually increased until electrical or mechanical failure occurred in the sample. During the measurement, a constant current of 100 mA was applied to the conductive pattern with a constant current power supply (PA36-2B, Texio Technology Co., Kanagawa, Japan). The voltage between the contact pads of the pattern was measured by a data logger (GL900-APS, Graphtec Co., Kanagawa, Japan) using the four-terminal method. The resistance of the pattern was calculated by dividing the measured voltage by the current of 100 mA. For cyclic tensile deformation, the deformation speed, elongation rate, and the number of elongation cycles were 165 mm/min, 10%, and 1000 cycles, respectively. During the measurement, the resistance change of the pattern was measured as in the single tensile deformation case.

## 3. Results and Discussion

### 3.1. Observation before Tensile Deformation

**Figure 4:**
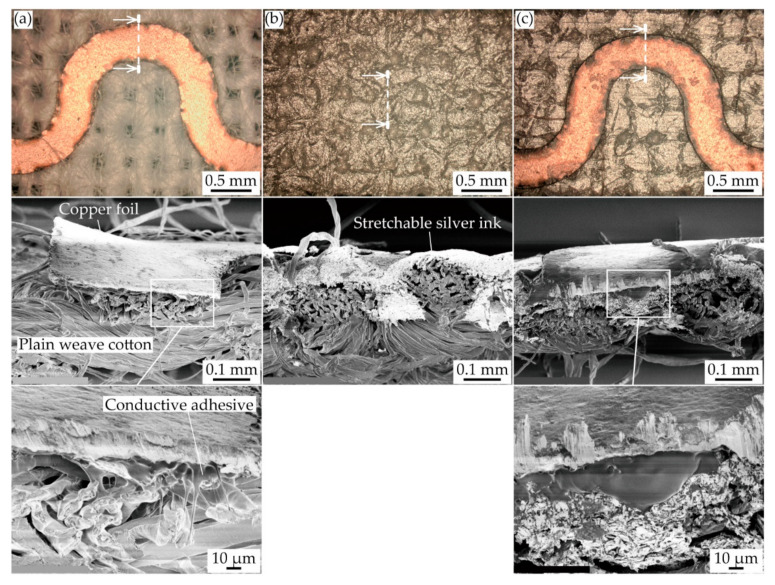
Optical and cross-sectional SEM images of (**a**) meandering copper; (**b**) silver ink; and (**c**) double-layered patterns.

Figure 4a–c show the optical and the cross-sectional SEM images of each type of conductive pattern. For the meandering copper patterns (Figure 4a), the images confirmed that the soft conductive adhesive between the pattern and the plain weave cotton permeated the cotton yarns. This indicates that the heat pressing during the fabrication process enhanced the mechanical adhesion between the pattern and plain weave cotton. For the silver ink patterns (Figure 4b), the silver ink also permeated into the yarns of the plain weave cotton. The effective thickness of the printed ink was measured from the cross-sectional SEM image, and the value was 0.07 mm. Therefore, the ratio of elongation stiffness of the silver ink patterns was 0.02. This indicates that multiple cracks should occur in the silver ink pattern. For the double-layered patterns (Figure 4c), the images confirmed that the soft conductive adhesive adhered between the copper and silver ink layers, indicating that the heat pressing during the fabrication process enhanced the electrical and mechanical adhesion between the copper and the silver ink layers.

### 3.2. Electrical Characterization under Single Tensile Deformation

**Figure 5:**
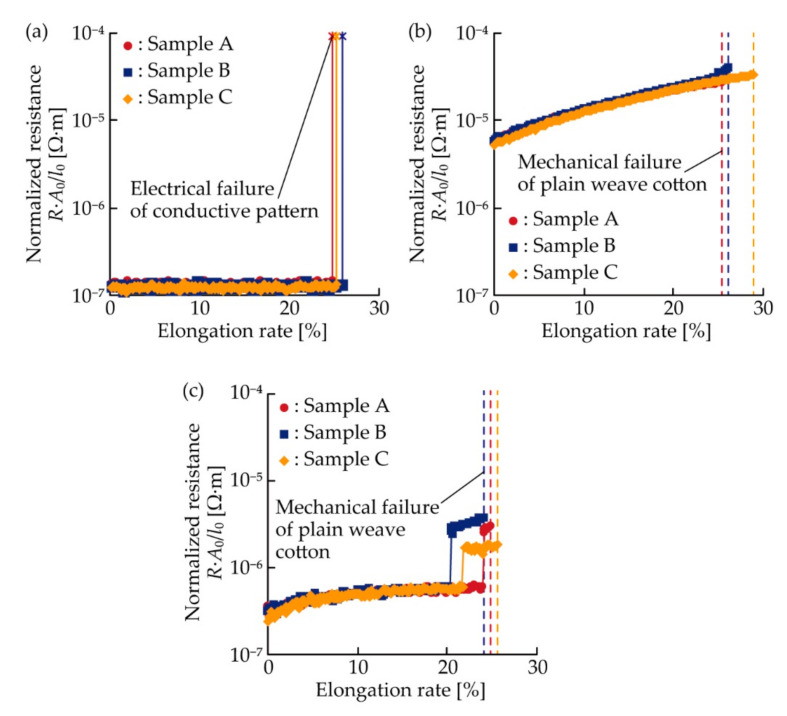
Normalized resistance plotted against the elongation rate for (**a**) meandering copper; (**b**) silver ink; and (**c**) double-layered patterns, under single tensile deformation.

Figure 5a–c show the resistance changes of each type of conductive pattern plotted against the elongation rate under single tensile deformation. The number of samples for each type was three (samples A, B, and C). The resistance was normalized to avoid dependence of comparison on the dimensions of the patterns. The normalization was conducted by producing the initial cross-sectional area, *A*_0_, and dividing it by the initial length, *l*_0_. *A*_0_ were 2 × 0.018, 2 × 0.07, and 2 × 0.088 mm^2^ for the meandering copper, silver ink, and double-layered patterns, respectively. *l*_0_ was 40 mm for each pattern. For the meandering copper patterns (Figure 5a), the resistances at the elongation rate of 0%, corresponding to the initial resistances, exhibited the lowest values (1.3 × 10^−7^ Ω·m) in each type of pattern. The resistances were almost constant, even when the elongation rate increased to 25%, and then increased sharply, indicating that electrical failure occurred in the patterns. These electrical characteristics are consistent with the characteristics of the pattern with single crack growth. In contrast, for the silver ink patterns (Figure 5b), the resistances increased gradually to 2.7, 3.9, and 3.3 × 10^−5^ Ω·m, for samples A, B, and C, respectively, as the elongation rate increased. Before the electrical failure of the conductive patterns, the plain weave cotton layers were mechanically broken. These electrical characteristics are consistent with the characteristics of conductive patterns with multiple crack growth. For the double-layered patterns (Figure 5c), the initial resistances exhibited lower values (3.0 × 10^−7^ Ω·m) than that of the silver ink patterns (5.5 × 10^−6^ Ω·m). When the elongation rate was low, the resistances increased slightly to 6.2, 5.6, and 6.5 × 10^−7^ Ω·m for samples A, B, and C, respectively, as the elongation rate increased. Then, the resistances increased shapely to 2.6, 2.9, and 1.7 × 10^−6^ Ω·m, respectively, and the resistances gradually increased again as the elongation rate increased. This indicates that the conductivity was maintained by the silver ink layer even though the electrical failures occurred in the copper layer. The plain weave cotton layers were also mechanically broken before the electrical failure of the double-layered patterns. Compared to the resistances of the meandering copper and silver ink patterns, the double-layered patterns maintained the lowest resistances, even at a high elongation rate.

**Figure 6:**
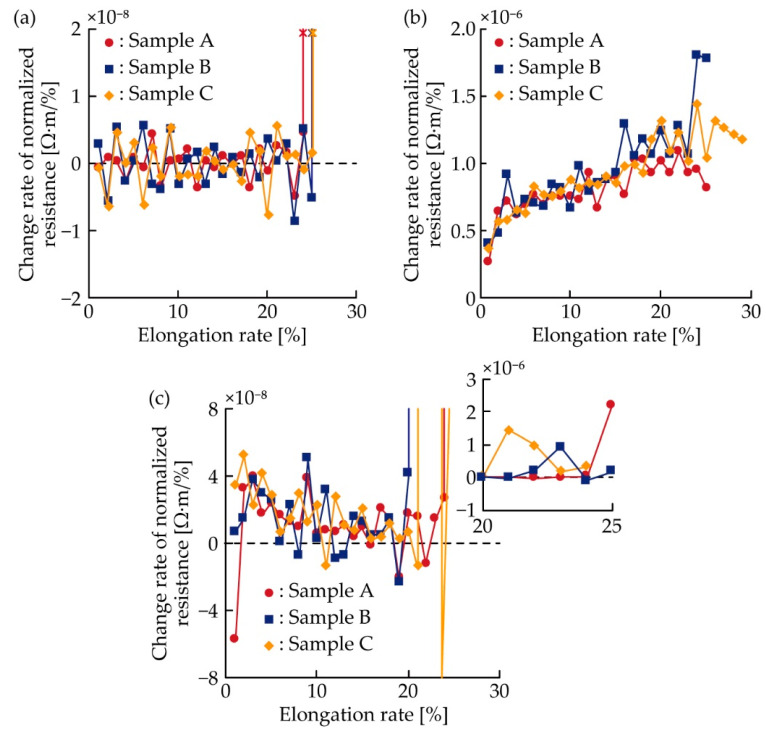
Change rate of the normalized resistance against the elongation rate of 1% in Figure 5a–c; (**a**) meandering copper; (**b**) silver ink; and (**c**) double-layered patterns.

For a better understanding of the resistance change rate under single tensile deformation, the change rate of the normalized resistance against the elongation rate, corresponding to 1% in Figure 5a–c, was analyzed, as shown in Figure 6a–c. The change rate was calculated from the average value of the normalized resistance at every elongation rate of 1%. For the meandering copper patterns (Figure 6a), although the change rates exhibited a fluctuation of approximately 10^−9^ Ω·m, the change rates were almost constant at zero. In contrast, for the silver ink patterns (Figure 6b), the change rates ranged on the order of 10^−7^ to 10^−6^ Ω·m, and increased gradually to approximately 1.0, 1.8, and 1.4 × 10^−6^ Ω·m, for samples A, B, and C, respectively, as the elongation rate increased. For the double-layered patterns (Figure 6c), the change rates decreased slightly of approximately 10^−8^ Ω·m when the elongation rate increased to approximately 20%. Then, the change rates increased sharply and ranged around 10^−7^ to 10^−6^ Ω·m. This sharp increase was caused by the electrical failure in the copper layer, and the change rates after the sharp increase were on the same order as that of the silver ink patterns. Therefore, the change rates of the meandering copper and silver ink patterns were constant, zero, and monotonically increased, respectively. In contrast, the change rate of the double-layered pattern varied considerably when the electrical failure occurred in the copper layer. The change rate after the failure was much higher than that before the failure, and on the same order as that of the silver ink pattern.

**Figure 7:**
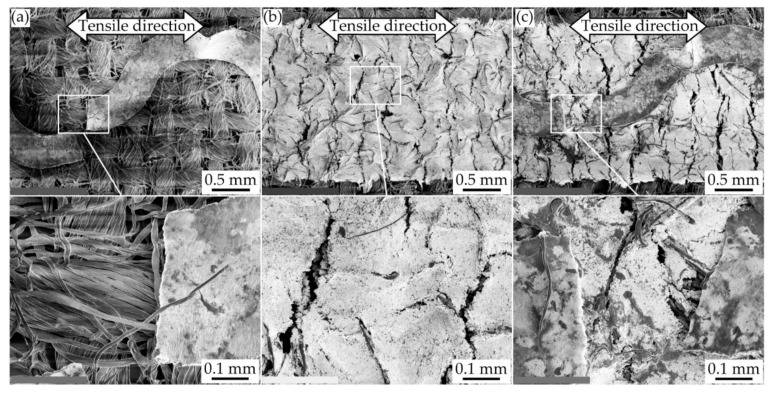
SEM images of conductive patterns after single tensile deformation; (**a**) meandering copper; (**b**) silver ink; and (**c**) double-layered patterns.

After the single tensile deformation, the cracks in each conductive pattern were observed by SEM, as shown in Figure 7a–c. For the meandering copper patterns (Figure 7a), the pattern was completely separated by the crack grown at one of the meander apexes. Other cracks were not observed around the separated point. This confirms that the resistance changes in Figure 5a were caused by the crack configuration of single crack growth. For the silver ink patterns (Figure 7b), many small cracks were observed in the hole of the pattern. The cracks propagated randomly in the tensile direction. However, complete separation of the pattern did not occur, confirming that the resistance changes in Figure 5b were caused by the crack configuration of multiple crack growth. For the double-layered patterns (Figure 7c), the copper layer was completely separated by the crack grown at one of the meander apexes, as in Figure 7a. Many small cracks were observed in the hole of the pattern, as in Figure 7b, but complete separation did not occur. This confirms that the resistance changes in Figure 5c were caused by the double-layered pattern with single and multiple crack growths.

### 3.3. Electrical Characterization under Cyclic Tensile Deformation

**Figure 8:**
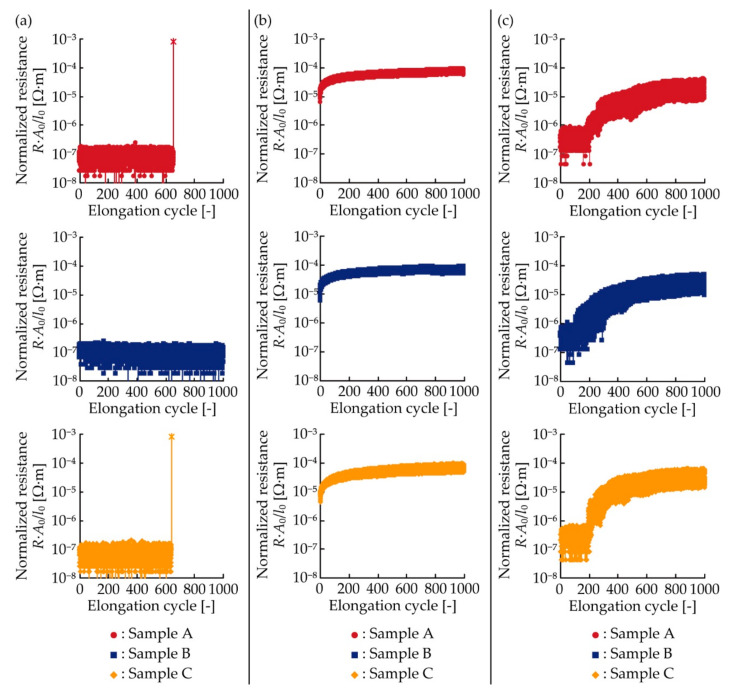
Normalized resistance plotted against elongation cycle for (**a**) meandering copper; (**b**) silver ink; and (**c**) double-layered patterns, under cyclic tensile deformation.

Figure 8a–c show the resistance changes of each type of conductive pattern plotted against the elongation cyclic under the cyclic tensile deformation. The number of samples for each type was also three (samples A, B, and C). The resistance was also normalized, as in Figure 5a–c, so that the comparison did not depend on the dimensions of the patterns. For the meandering copper patterns (Figure 8a), although the fluctuation on the order of 10^−8^ Ω·m occurred, the resistances were almost constant even under the cyclic tensile deformation of 10%. However, the resistances of samples A and C increased sharply when the elongation cyclic was 655 and 638 cycles, respectively. This indicates that electrical failure occurred in the patterns. These electrical characteristics are also consistent with the characteristics of the patterns with single crack growth. For the silver ink patterns (Figure 8b), the range of resistances increased gradually and shifted to the higher values as the elongation cycle increased. These electrical characteristics are also consistent with the characteristics of the patterns with multiple crack growth. For the double-layered patterns (Figure 8c), the resistances were almost constant in the range of approximately 10^−7^ Ω·m when the elongation cycle was under approximately 200 cycles. The resistances then gradually increased as the elongation rate increased, indicating that the electrical failure occurred in the copper layer. Compared to the resistances of the meandering copper and silver ink patterns, the resistance of the double-layered pattern maintained the smallest resistance, even at the high elongation cycle.

**Figure 9:**
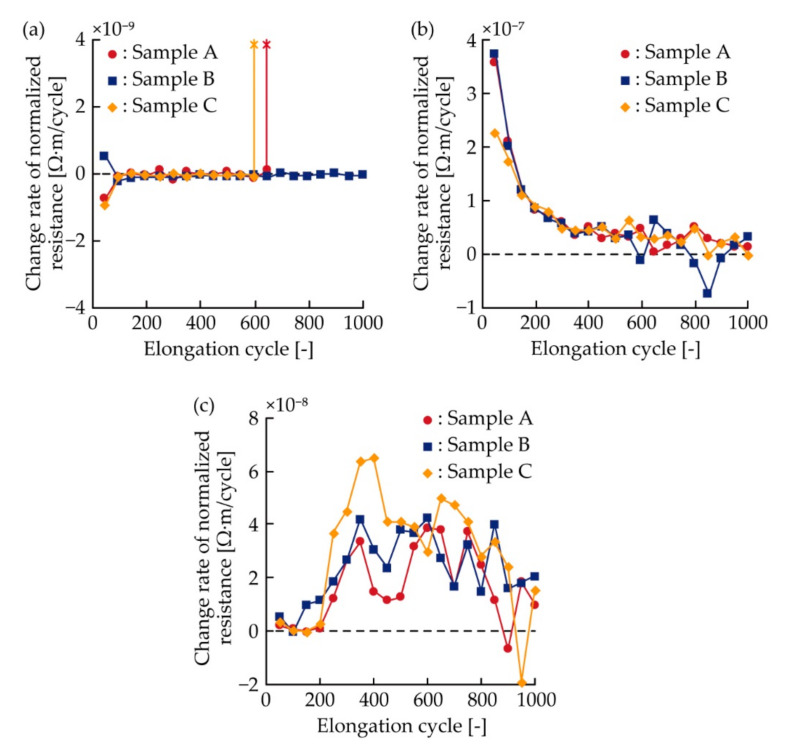
Change rate of normalized resistance against the elongation cycle of one cycle in Figure 8a–c; (**a**) meandering copper, (**b**) silver ink, and (**c**) double-layered patterns.

For a better understanding of the resistance change under cyclic tensile deformation, the change rate of the normalized resistance against the elongation cycle corresponding to one cycle in Figure 8a–c was also analyzed, as shown in Figure 9a–c. The change rate was calculated from the average value of the normalized resistance at every 50 cycles. For the meandering copper patterns (Figure 9a), the change rates were almost constant at zero. For the silver ink patterns (Figure 9b), the change rates decreased as the elongation cyclic increased to approximately 400 cycles. Then, the change rates were almost constant, approximately 6 × 10^−8^ Ω·m. This asymptote might have been caused by stress softening because of the Mullins effect in the silver ink [30,31], and friction between the yarns in the plain weave cotton [32]. For the double-layered patterns (Figure 9c), the change rates were almost constant on the order of 10^−9^ Ω·m, when the cyclic number was under approximately 200 cycles. Then, the change rates repeated the sharp increase and decrease on the order of 10^−8^ Ω·m. The increase should be caused by the electrical failure in the copper layer, and the sharp decrease should be caused by the stress softening in the silver ink layer. Therefore, the change rates of the meandering copper and silver ink patterns were constant, zero, and monotonically decreased, respectively. In contrast, that of the double-layered pattern varied considerably when the electrical failure occurred in the copper layer. The change rate after the failure were much higher than that before the failure and on the same order as that of the silver ink pattern.

**Figure 10:**
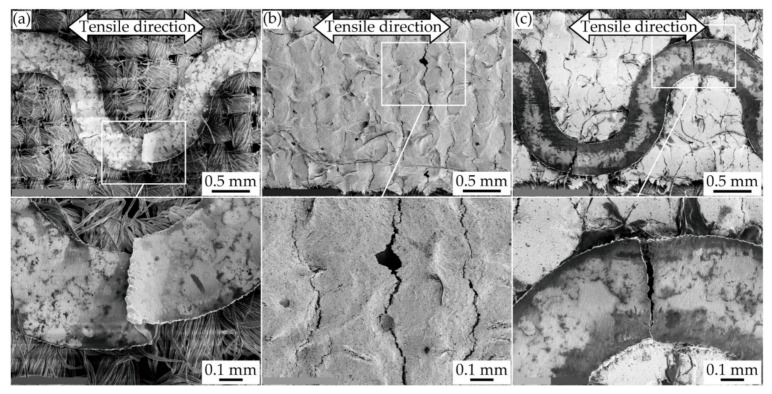
SEM image of conductive patterns after cyclic tensile deformation; (**a**) meandering copper, (**b**) silver ink; and (**c**) double-layered patterns.

After the cyclic tensile deformation, the cracks in each conductive pattern were also observed by SEM, as shown in Figure 10a–c. For the meandering copper patterns (Figure 10a), the pattern was completely separated by the crack grown at one of the meander apexes. This also confirms that the resistance changes in Figure 8a were caused by the crack configuration of single crack growth. For the silver ink patterns (Figure 10b), several small cracks were observed in the hole of the pattern, and the complete separation of the pattern did not occur. This also confirms that the resistance changes in Figure 8b were caused by the crack configuration of multiple crack growth. For the double-layered patterns (Figure 10c), single and multiple crack growths were observed in the copper and silver ink layers, respectively, and some complete separations were observed at the meander apexes. This confirms that the resistance change in Figure 8c was caused by the double-layered patterns with single and multiple crack growths.

## 4. Conclusions

The double-layered conductive pattern consisting of patterns with single and multiple crack configurations was investigated. Each layer was theoretically designed to control the crack configuration, and then the meandering copper pattern, silver ink patterns, and their double-layered patterns were fabricated on plain weave cottons as patterns with single and multiple crack growth and double-layered patterns, respectively. Their resistance changes under a single (large) and cyclic tensile deformation were characterized. The results showed that the double-layered pattern maintained the lowest resistance at the high elongation rate and cycle. The resistance change rates of the meandering copper and silver ink patterns were constant and monotonically changed against the elongation rate/cycle, respectively. In contrast, the change rate of the double-layered pattern varied considerably when the electrical failure occurred in the copper layer. The change rate after the failure was much higher than that before the failure, and on the same order as that of the silver ink patterns. This study could lay the foundations for developing a double-layered pattern for the conductive patterns of e-textiles.
